# Watchful Waiting Phase As Window of Opportunities for Non-pharmaceutical and Non-surgical Management of a Small Splenic Cyst Complicated With Thrombocytopenia

**DOI:** 10.7759/cureus.48817

**Published:** 2023-11-14

**Authors:** Xiao Wang, Yanzhi Wang

**Affiliations:** 1 Medical Education and Simulation, Ascentin LLC, New York, USA; 2 Surgery and Anatomy, Xiangya Medical College, Changsha, CHN

**Keywords:** dietary intervention, watchful waiting, management, non-pharmaceutical, non-surgical, surgery, complication, spleen, thrombocytopenia, splenic cyst

## Abstract

An asymptomatic splenic cyst smaller than 50 mm was detected incidentally at a routine health checkup. Management of the cyst, affected and determined by multiple factors, including force majeure, became difficult and thrombocytopenia developed during watchful waiting. Spontaneous recovery of the spleen did not occur with continued watchful waiting, and thrombocytopenia worsened. However, when a three-month dietary intervention was subsequently implemented, the initiation of recovery was observed. The diet modification was adding to regular meals a daily serving of vegetables prepared following traditional Chinese culinary style. A second course of dietary intervention was undertaken, and accelerated recovery was detected thereafter, with eventual complete resolution of the splenic cyst and thrombocytopenia. This case demonstrates the feasibility and potential benefits of lifestyle intervention for the management of small splenic cysts, including those complicated with thrombocytopenia. Lifestyle intervention, such as dietary intervention, is particularly suitable for the watchful waiting phase since disease management during this time is non-pharmaceutical and non-surgical by nature.

## Introduction

There are currently no evidence-based consensus systematic guidelines available for the management of splenic cysts, which are rare conditions [1-3]. It is unlikely that the situation will change soon as large-scale randomized clinical studies are not always considered practically feasible for diseases of low prevalence. 

Case management becomes understandably challenging for healthcare practitioners who are faced with splenic cysts since most are discovered incidentally [1,2]. Surgery has been the treatment of choice for active splenic cyst management, with splenectomies being the mainstay [1,2]. Although splenectomies can produce curative outcomes, the risks of surgical and post-operative complications [4,5] have prompted the emergence of two notable developments in care: (1) the introduction of less invasive and more spleen-sparing surgical procedures [5] and (2) the advocation of watchful waiting [2], an approach that both clinicians and patients may find appealing upon first detection of minor asymptomatic cysts.

The criteria for surgical intervention are inconsistent and convoluted. Traditionally, the cutoff for the recommendation of splenectomy is a cyst size of 50 mm [2-4]. The 50 mm threshold, or any size threshold, has been challenged by a study conducted by Kenney et al. that found cyst size to be irrelevant in this regard [6]. 

On the other hand, a monitoring approach is not necessarily optimal, even for small splenic cysts, because there may be undetected complications that would remain unaddressed. 

Complications, whether detectable or not, make the management of splenic cysts much more challenging for practitioners as they seek a balance between treating the disease and sparing the spleen and its functions. 

One notable complication of splenic cysts is thrombocytopenia [5]. Thrombocytopenia is managed either pharmaceutically or surgically, and primary and secondary thrombocytopenia can be treated similarly with respect to methodology [7]. Generally, splenectomy is indicated for splenic cysts, even if their size is smaller than 50 mm, when complicated with thrombocytopenia [3,4]. The consideration may be justified as the spleen is prone to rupture [8], and a ruptured spleen coupled with thrombocytopenia imposes a serious risk of bleeding. 

Nevertheless, it is still possible to avoid surgery. For instance, thrombocytopenia may be addressed with medication [7], while the splenic cyst is monitored regularly [2]. The strategy becomes less favorable when the splenic cyst is the underlying cause of thrombocytopenia. Whether the conditions of minor cysts and thrombocytopenia can be properly managed while also sparing the spleen remains an open question. 

In this report, we present a case of a small splenic cyst with the complication of thrombocytopenia. For reasons explained below, the patient was managed non-pharmaceutically and non-surgically. Satisfactory outcomes were eventually achieved.

## Case presentation

A small splenic cyst was detected on ultrasound in a Chinese female in her 30s with no prior history of splenic cysts at a routine health checkup in a Chinese town (year one). Upon detection, she was asymptomatic, and it was determined that she would proceed only with regular monitoring.

**Figure 1:**
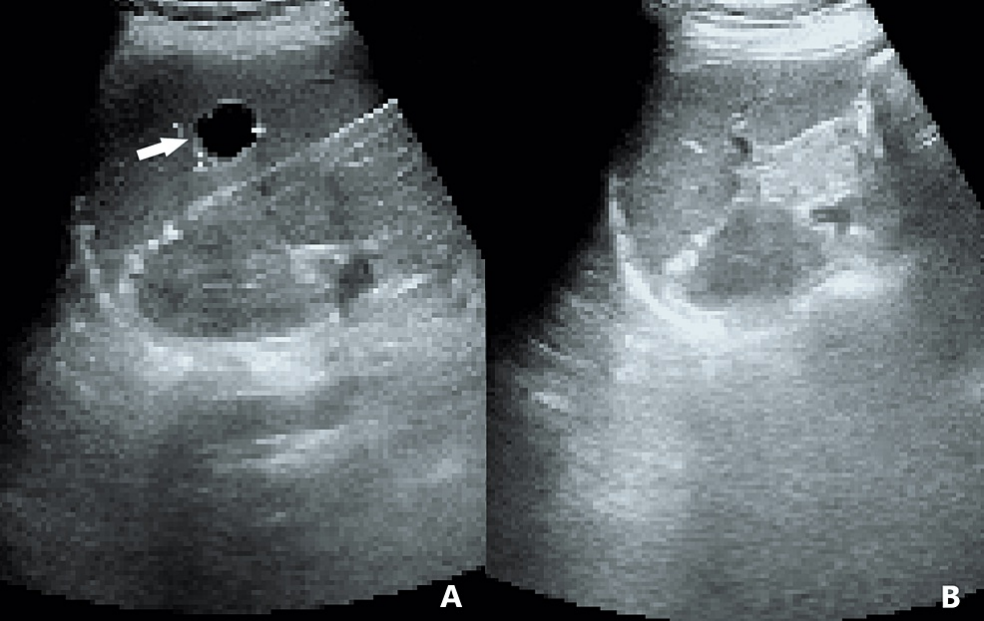
Ultrasound scans of the spleen of the patient before and after dietary intervention. (A) Sonogram showing a splenic cyst (arrow) measuring 18.9 mm x 17 mm in year one. (B) No cyst can be seen on the ultrasound scan in year six after two courses of dietary intervention, which were carried out in year three and year six, respectively.

Ten months later, she received another examination, and an 18.9 mm x 17 mm splenic cyst was confirmed by ultrasonography (Figure 1A). She also developed thrombocytopenia (platelet count at 94 x 10^9^/L), a complication that was not present on the initial detection of the splenic cyst. Thrombocytopenia in Chinese, as defined by national guidelines, is the condition when the platelet count is lower than 100 x 10^9^/L [9]. She did not receive any treatment.

In year two, the splenic cyst remained stable, but her platelet count declined further to 72 x 10^9^/L. Early in year three, in light of the outcomes of year two, it was thought that a proactive approach taken before surgery might offer more benefit than continuous monitoring alone. Furthermore, it was probably more desirable to address the splenic cyst rather than directly treating thrombocytopenia with medication, as the former was likely the underlying cause of the latter in her case.

Taking into account the factor of force majeure at that time, the patient opted for dietary intervention after we provided her with a thorough discussion. The decision for dietary intervention was made based on the consideration that vegetables have anti-inflammatory phytochemicals [10], which could potentially benefit her spleen. She was given a daily course of various seasonal vegetables prepared from 150 g of fresh materials and cooked following traditional Chinese culinary style. It was an extra course of food taken in addition to her regular meals. The dietary intervention ended after three months due to force majeure.

Thereafter, she underwent her regular health examination for year three. Interestingly, after the non-pharmaceutical, dietary intervention, the size of the splenic cyst decreased from 18 mm x 17 mm to 18 mm x 15 mm, and the platelet count recovered from 72 x 10^9^/L to 86 x 10^9^/L, when compared with a year ago.

In year four, without additional treatment and when compared to the prior year, the size of the splenic cyst dropped from 18 mm x 15 mm to 15 mm x 14 mm and the platelet count remained stable. In year five, when compared to year four, the splenic cyst shrank considerably from 15 mm x 14 mm to 12 mm x 10 mm and thrombocytopenia resolved (platelet count at 108 x 10^9^/L).

The dietary intervention was resumed in year six, immediately when it became available, and continued for four months until her regular checkup. Following this course of dietary intervention, the splenic cyst became undetectable on ultrasound (Figure 1B), and the platelet count rose further to 133 x 10^9^/L (Table 1).

**Table 1:** Splenic cyst size and platelet count of the patient over the six years since the cyst was found incidentally and until the second course of dietary intervention was completed.

Time	Splenic cyst size (mm^2^)	Platelet count (x 10^9^/L)
February 2018 (year one)	A cyst detected	134
December 2018 (year one)	18.9 x 17	94
November 2019 (year two)	18 x 17	72
September 2020 (year three)	18 x 15	86
September 2021 (year four)	15 x 14	84
September 2022 (year five)	12 x 10	108
September 2023 (year six)	0 x 0	133

Throughout the six years following the initial diagnosis of the splenic cyst, the patient experienced no additional symptoms associated with the cyst other than thrombocytopenia; she was not hospitalized, nor was her job performance affected.

## Discussion

On initial detection, the current case appeared to be a typical minor splenic cyst. However, the progression of the disease and its management, as we recorded, showcased an interesting course and outcome.

Although the patient exhibited no symptoms or complications upon initial detection of the splenic cyst, she did not show spontaneous recovery and, instead, developed thrombocytopenia after 10 months during watchful waiting. Consequently, with the emergence of thrombocytopenia, surgery was indicated based on accepted expert opinion, although individualized management was encouraged [3]. Decision-making became more challenging when surgery was not practically feasible at that point in time.

As watchful waiting continued, spontaneous improvement of the splenic cyst still did not occur, and the platelet count kept deteriorating. 

With the situation affected and determined by multiple factors collectively, we provided the option of dietary intervention to the patient. Early signs of recovery appeared after the first course of dietary intervention, with the initiation of platelet count rebound and cessation of splenic cyst growth. Data from the following two years, i.e., years four and five, confirmed that a recovery process was initiated. Surgery was therefore no longer in consideration. The observation led to the decision to resume the dietary intervention as soon as it became available. Accelerated recovery was seen after the second course of dietary intervention, and a complete resolution of disease and complications ultimately ensued. 

Here we have reported the sequence of events of the case from first detection to resolution, chronologically, with the intention of adding to the current knowledge of the field. It would be very interesting to see if larger splenic cysts could be managed similarly.

## Conclusions

The case demonstrates the feasibility and potential benefits of lifestyle intervention for the management of small splenic cysts, including those complicated with thrombocytopenia. This non-pharmaceutical and non-surgical approach is particularly suitable for the watchful waiting phase of disease management. 
